# Supplementary material for: End Sequence Analysis Toolkit (ESAT) expands the extractable information from single-cell RNA-seq data
Source: Genome Res. 2016 Oct;26(10):1397–410. doi: 10.1101/gr.207902.116 (PMC5052061; doi:10.1101/gr.207902.116)
Supplement: Supplemental Material [file supp_gr.207902.116_Supp_Materials.tar.gz › ESAT-esat_v0.1/doc/html/functions_m.html]

ESAT: Class Members


ESAT


- Main Page
- Related Pages
- Packages
- Classes
- Files

- Class List
- Class Index
- Class Hierarchy
- Class Members

- All
- Functions
- Variables
- Enumerator

- a
- b
- c
- d
- e
- f
- g
- h
- i
- j
- k
- l
- m
- n
- o
- p
- q
- r
- s
- t
- u
- v
- w
- z


All Classes Namespaces Files Functions Variables Enumerator Pages

Here is a list of all class members with links to the classes they belong to:

### - m -

- MAD()
  : broad.core.math.Statistics
- MAFAlignment()
  : broad.core.multiplealignment.MAFAlignment
- MAFIO()
  : broad.core.multiplealignment.MAFIO
- main()
  : broad.core.annotation.AnnotationUtils
  , broad.core.annotation.MaximumContiguousSubsequence
  , broad.core.datastructures.MatrixWithHeaderUtils
  , broad.core.math.ANOVA
  , broad.core.math.CombinationGenerator
  , broad.core.math.EmpiricalDistribution
  , broad.core.math.FDRDistribution
  , broad.core.math.Loess
  , broad.core.math.PermutationDistribution
  , broad.core.math.Statistics
  , broad.core.motif.misc.ComputeConservedMotifs
  , broad.core.motif.misc.ScoreMotifInstances
  , broad.core.motif.PositionWeightMatrixIO
  , broad.core.motif.PWMUtils
  , broad.core.motif.SequenceMotif
  , broad.core.motif.SequenceMotifCluster
  , broad.core.multiplealignment.AlignmentExtractor
  , broad.core.multiplealignment.MAFAlignment
  , broad.core.multiplealignment.MultipleAlignmentFactory
  , broad.core.overlaputils.GeneSetIntersect
  , broad.core.primer3.ComputeMIRScore
  , broad.core.primer3.isPCRLike
  , broad.core.primer3.PrimerUtils
  , broad.core.sequence.Extractor
  , broad.core.sequence.FastaQualityIndex
  , broad.core.sequence.FastaSequenceIO
  , broad.core.sequence.TranscribedRegions
  , broad.core.sequence.TranscribedSequenceComposition
  , broad.core.siphy.PiHMM
  , broad.core.siphy.tools.conservation.AverageBranchLength
  , broad.core.siphy.tools.conservation.BatchSubmitEstimateExon
  , broad.core.siphy.tools.conservation.ComputeKmersForRegion
  , broad.core.siphy.tools.conservation.ComputeMaxPercentileOfElements
  , broad.core.siphy.tools.conservation.ComputePercentageOfKmers
  , broad.core.siphy.tools.conservation.EstimateOmegaPerExon
  , broad.core.siphy.tools.conservation.SplitFile
  , broad.core.siphy.TreeScaler
  , broad.pda.annotation.BEDFileParser
  , broad.pda.countreads.AnnotationCounts
  , broad.pda.countreads.FastqLibraryStats
  , broad.pda.differentialExpression.BatchNorm
  , broad.pda.differentialExpression.CisVsTrans
  , broad.pda.differentialExpression.CollapseProjections
  , broad.pda.differentialExpression.ConvertToDiscreteMatrix
  , broad.pda.differentialExpression.FixedControlDifferentialExpression
  , broad.pda.differentialExpression.FoldSignificance
  , broad.pda.differentialExpression.GrabProbes
  , broad.pda.differentialExpression.GSAProjection
  , broad.pda.differentialExpression.MakePositionalGeneSets
  , broad.pda.differentialExpression.MergeGSEAOutputIntoMatrix
  , broad.pda.differentialExpression.RunAllGSEABatch
  , broad.pda.differentialExpression.RunDifferentialExpression
  , broad.pda.differentialExpression.RunGSEA
  , broad.pda.differentialExpression.RunTimeSeries
  , broad.pda.differentialExpression.SplitByName
  , broad.pda.gene.functionalAnnotation
  , broad.pda.gene.GeneTools
  , broad.pda.gene.NeighborAnalysis
  , broad.pda.geneexpression.agilent.AgilentToGCT
  , broad.pda.geneexpression.agilent.analysis.ProcessDataset
  , broad.pda.geneexpression.agilent.ClassifyAgilentLincProbes
  , broad.pda.geneexpression.agilent.CollapseProbesByMinPValue
  , broad.pda.geneexpression.agilent.CorrelationAgilentNanostring
  , broad.pda.geneexpression.agilent.CorrelationBetweenReplicates
  , broad.pda.geneexpression.agilent.ExtractSamplesAndLabel
  , broad.pda.geneexpression.agilent.FilterMatrixByMinCutoff
  , broad.pda.geneexpression.agilent.FindProbesForGeneSet
  , broad.pda.geneexpression.agilent.MakeCLSFiles
  , broad.pda.geneexpression.agilent.MakeCLSFromDescription
  , broad.pda.geneexpression.agilent.QCAgilentResults
  , broad.pda.geneexpression.clustering.ComputeCorrelationOfReplicates
  , broad.pda.geneexpression.clustering.HierarchicalClustering
  , broad.pda.geneexpression.clustering.RankOrder
  , broad.pda.geneexpression.ComputeHypergeomtricEnrichment
  , broad.pda.geneexpression.CountNums
  , broad.pda.geneexpression.FilterGCTFile
  , broad.pda.geneexpression.geneExpUtils
  , broad.pda.geneexpression.GlobalStats
  , broad.pda.geneexpression.GrabSpecificColumns
  , broad.pda.geneexpression.PaGEJava
  , broad.pda.geneexpression.PreprocessDataSet
  , broad.pda.rap.misc.FindEnrichedDepletedRegions
  , broad.pda.rnai.ExtractSequence
  , broad.pda.samtools.FilterFastqReads
  , broad.pda.samtools.FilterPairedReadsByMAQ
  , broad.pda.samtools.PairedEndTDF
  , broad.pda.samtools.SAMFragmentWriter
  , broad.pda.samtools.TrimFastqReads
  , broad.pda.seq.alignment.AlignmentUtils
  , broad.pda.seq.chip.ComputeEnrichments
  , broad.pda.seq.chip.FindDiffrentialEnrichedRegions
  , broad.pda.seq.chip.PeakFilter
  , broad.pda.seq.clip.FastaExtendPeaks
  , broad.pda.seq.clip.Pileup
  , broad.pda.seq.fastq.FastqSplitterByBarcode
  , broad.pda.seq.fastq.SplitAndTrimIndex
  , broad.pda.seq.fastq.SplitFastqByIndex
  , broad.pda.seq.protection.BatchedMultiSampleScanPeakCaller
  , broad.pda.seq.protection.MultiSampleScanPeakCaller
  , broad.pda.seq.protection.ReadSimulator
  , broad.pda.seq.protection.ReadSimulator2
  , broad.pda.seq.rap.BuildRatioNullDistribution
  , broad.pda.seq.rap.CalculateCoverageStats
  , broad.pda.seq.rap.CollectAnnotationEnrichments
  , broad.pda.seq.rap.CountProbes
  , broad.pda.seq.rap.CountReads
  , broad.pda.seq.rap.DistanceToBED
  , broad.pda.seq.rap.GCContent
  , broad.pda.seq.rap.PlotAggregateRegions
  , broad.pda.seq.rap.RAPPeakCaller
  , broad.pda.seq.rap.RatioPermutationPeakCaller
  , broad.pda.seq.rap.rna.CollectRnaSeqMetrics
  , broad.pda.seq.rap.rna.CountReads
  , broad.pda.seq.rap.ShuffleBAM
  , broad.pda.seq.rap.ShuffleBED
  , broad.pda.seq.rap.SkellamKLPeakCaller
  , broad.pda.seq.rap.SlideAndCalculate
  , broad.pda.seq.rap.SlideAndCount
  , broad.pda.seq.rap.UnmarkDuplicates
  , broad.pda.seq.segmentation.ContinuousDataAlignmentModel
  , broad.pda.seq.segmentation.GlobalSpliceFilter
  , broad.pda.seq.segmentation.PrecomputeDataAlignmentStats
  , broad.pda.seq.segmentation.utils.ReconstructionComparer
  , broad.pda.tools.PSLXToSAM
  , broad.projection.gsa.DefineGeneSignatures
  , broad.projection.gsa.ExtractGenes
  , broad.projection.gsa.GSAScoring
  , broad.projection.gsa.GsaUtils
  , umms.core.alignment.FeatureSequenceAlignment
  , umms.core.alignment.SmatchLike
  , umms.core.annotation.BasicAnnotation
  , umms.core.model.JCSAlignmentModel
  , umms.core.readers.PairedEndReader
  , umms.core.scripture.BuildScriptureCoordinateSpace
  , umms.core.scripture.statistics.ConnectDisconnectedTranscripts
  , umms.esat.CollapsedGeneLengths
  , umms.esat.ESAT
  , umms.esat.ESATDistanceCalculator
- MAIN\_ARRAY\_LEFT
  : broad.pda.seq.rap.CountProbes
- MAIN\_ARRAY\_RIGHT
  : broad.pda.seq.rap.CountProbes
- makeBinnedIntervalModel()
  : broad.pda.gene.NeighborsNullModel
- MakeCLSFiles()
  : broad.pda.geneexpression.agilent.MakeCLSFiles
- MakeCLSFromDescription()
  : broad.pda.geneexpression.agilent.MakeCLSFromDescription
- makeDartThrowingModel()
  : broad.pda.gene.NeighborsNullModel
- makeDataMatrix()
  : broad.pda.geneexpression.agilent.AgilentUtils
- makeGenes()
  : broad.pda.annotation.BEDFileParser
- makeIntervalTree()
  : broad.core.util.CollapseByIntersection
- makeIntervalTreeFor3Prime()
  : broad.pda.annotation.BEDFileParser
- makeIntervalTreeForGeneExons()
  : broad.core.util.CollapseByIntersection
- makeIntervalTreeForGenes()
  : broad.core.util.CollapseByIntersection
- makeNaiveRandModel()
  : broad.pda.gene.NeighborsNullModel
- makePairs()
  : umms.core.alignment.AlignmentPair
- MakePositionalGeneSets()
  : broad.pda.differentialExpression.MakePositionalGeneSets
- makeRandTranscriptList()
  : broad.pda.gene.transcriptsNullModel
- manhattanDistance()
  : broad.core.math.Statistics
- MannWhitney()
  : broad.core.math.MannWhitney
- MannWhitneyPermutations()
  : broad.core.math.MannWhitneyPermutations
- mapChildToLargestParent()
  : umms.core.utils.AnnotationUtils
- mapChildToParents()
  : umms.core.utils.AnnotationUtils
- mapGenesToOverlappers()
  : broad.core.overlaputils.GeneSetIntersect
- mapParentsToChildren()
  : umms.core.utils.AnnotationUtils
- MappingQualityFilter()
  : umms.core.readFilters.MappingQualityFilter
- mapSamRecord()
  : broad.pda.seq.protection.ReadSimulator
  , broad.pda.seq.protection.ReadSimulator2
- MarkovModel()
  : broad.core.hmm.MarkovModel< T >
- mask()
  : broad.core.sequence.Sequence
- MASK\_FILE
  : broad.pda.seq.rap.GenomeCommandLineProgram
- MaskedGenomicSpace()
  : umms.core.coordinatesystem.MaskedGenomicSpace
- maskSoftmaskedRegions()
  : broad.core.sequence.Sequence
- match()
  : broad.core.motif.PositionWeightMatrix
  , broad.core.motif.SequenceMotif
  , broad.core.motif.SequenceMotifCluster
  , umms.core.annotation.PSLRecord
- MATCH\_OPPOSITE\_STRAND
  : broad.pda.seq.rap.DistanceToBED
- matchStartOnFirstSequence()
  : umms.core.alignment.SmithWatermanAlignment
- mateCigarFlag
  : umms.core.writers.PairedEndWriter
- mateLineFlag
  : umms.core.writers.PairedEndWriter
- mateSequenceFlag
  : umms.core.writers.PairedEndWriter
- Matrix()
  : jaligner.matrix.Matrix
  , Jama.Matrix
- MatrixLoaderException()
  : jaligner.matrix.MatrixLoaderException
- MatrixWithHeaders()
  : broad.core.datastructures.MatrixWithHeaders
- max()
  : broad.core.datastructures.IntervalTree< V >
  , broad.core.math.MathUtil
  , broad.core.math.Statistics
- MAX\_DISTANCE
  : umms.core.annotation.AbstractAnnotation
- MAX\_FRAGMENT\_LENGTH
  : broad.pda.seq.rap.GenomeCommandLineProgram
- MAX\_INSERT
  : broad.pda.samtools.PairedEndTDF
  , broad.pda.samtools.SAMPairedEndFileReader
- maxDist
  : broad.pda.differentialExpression.FWERDistribution
- maxFragmentLengthData
  : broad.pda.seq.clip.SampleData
  , broad.pda.seq.protection.SampleData
- maxFragmentLengthGeneScores
  : broad.pda.seq.clip.SampleData
- maxInt()
  : broad.core.math.Statistics
- maxMAD()
  : broad.core.math.Statistics
- maxmean()
  : broad.core.math.Statistics
- maxSelfComplementarity()
  : broad.core.primer3.HairpinKmer
- maxStdev()
  : broad.core.math.Statistics
- maxSubSum3()
  : broad.core.annotation.MaximumContiguousSubsequence
- mayHaveBlocks()
  : broad.core.annotation.BasicGenomicAnnotation
  , broad.core.annotation.BED
  , broad.core.annotation.GenomicAnnotation
  , broad.core.sequence.SequenceRegion
- mean()
  : broad.core.math.Statistics
- meanNormalizeColumns()
  : broad.core.datastructures.MatrixWithHeaders
- meanNormalizeRows()
  : broad.core.datastructures.MatrixWithHeaders
- measure()
  : broad.pda.geneexpression.clustering.AbsolutePearsonFunction
  , broad.pda.geneexpression.clustering.ClusterDistanceFunction
  , broad.pda.geneexpression.clustering.EuclideanDistance
  , broad.pda.geneexpression.clustering.PearsonDistance
- median()
  : broad.core.math.Statistics
  , broad.pda.differentialExpression.BatchNorm
- medianCenterColumns()
  : broad.core.datastructures.MatrixWithHeaders
- medianCenterColumnsThis()
  : broad.core.datastructures.MatrixWithHeaders
- medianCenterRows()
  : broad.core.datastructures.MatrixWithHeaders
- medianCenterRowsThis()
  : broad.core.datastructures.MatrixWithHeaders
- medianCollection()
  : broad.core.math.Statistics
- medianNormalizeColumns()
  : broad.core.datastructures.MatrixWithHeaders
- medianNormalizeRows()
  : broad.core.datastructures.MatrixWithHeaders
- MemeMotif()
  : broad.core.motif.meme.MemeMotif
- MemeResultReader()
  : broad.core.motif.meme.MemeResultReader
- memoryStats()
  : umms.core.scripture.BuildScriptureCoordinateSpace
- merge()
  : broad.core.annotation.AnnotationReader< T extends GenomicAnnotation >
  , broad.pda.annotation.BEDFileParser
  , broad.pda.datastructures.Alignments
  , broad.pda.gene.GeneTools
- mergeAllOverlappers()
  : broad.core.annotation.BasicLightweightAnnotation
- mergeByGtfGeneId()
  : broad.pda.annotation.BEDFileParser
  , broad.pda.gene.GeneTools
- MergeGSEAOutputIntoMatrix()
  : broad.pda.differentialExpression.MergeGSEAOutputIntoMatrix
- mergeOverlappingBlocks()
  : umms.core.utils.AnnotationUtils
- mergeOverlappingBlocksSingleOrientation()
  : umms.core.utils.AnnotationUtils
- mergePeaks()
  : broad.pda.seq.protection.MultiSampleScanPeakCaller
- mergeRecords()
  : broad.core.sequence.FastaSequenceIO
- mergeSets()
  : broad.pda.gene.GeneTools
- metaAvg()
  : broad.core.math.Statistics
- METRICS
  : broad.pda.samtools.FilterPairedReadsByMAQ
- MIDDLE\_LENGTH
  : broad.pda.seq.rap.PlotAggregateRegions
- min()
  : broad.core.datastructures.IntervalTree< V >
  , broad.core.math.Statistics
- MIN\_MAPPING\_QUALITY
  : broad.pda.seq.rap.GenomeCommandLineProgram
- MIN\_WINDOW\_COUNT
  : broad.pda.seq.protection.TwoSampleScanSkellamPeakCaller
- minDist
  : broad.pda.differentialExpression.FWERDistribution
- MINIMUM\_MAPPING\_QUALITY
  : broad.pda.samtools.FilterPairedReadsByMAQ
- minInt()
  : broad.core.math.Statistics
- minOverlapper()
  : broad.core.datastructures.IntervalTree< V >
- minus()
  : broad.core.annotation.AnnotationReader< T extends GenomicAnnotation >
  , broad.core.datastructures.MatrixWithHeaders
  , broad.core.sequence.SequenceRegion
  , broad.pda.annotation.BEDFileParser
  , Jama.Matrix
  , umms.core.annotation.Annotation
  , umms.core.annotation.AnnotationList< T extends Annotation >
  , umms.core.annotation.BasicAnnotation
  , umms.core.annotation.CompoundInterval
- minusEquals()
  : Jama.Matrix
- MISMATCH
  : jaligner.Markups
- MmeI
  : broad.core.primer3.RestrictionEnzyme
- moveAnnotation()
  : umms.core.coordinatesystem.TranscriptomeSpace
- moveToCoordinate()
  : broad.core.sequence.SequenceRegion
  , umms.core.alignment.AbstractPairedEndAlignment
  , umms.core.annotation.Annotation
  , umms.core.annotation.BasicAnnotation
  , umms.core.annotation.CompoundInterval
- MRNA\_ARRAY\_LEFT
  : broad.pda.seq.rap.CountProbes
- MRNA\_ARRAY\_RIGHT
  : broad.pda.seq.rap.CountProbes
- MultipleAlignment()
  : broad.core.multiplealignment.MultipleAlignment
- multiplyColumnsWithConstants()
  : broad.core.datastructures.MatrixWithHeaders
- MultiSampleScanPeakCaller()
  : broad.pda.seq.protection.MultiSampleScanPeakCaller
- MultiScore()
  : umms.core.model.score.MultiScore


---

Generated on Fri Aug 15 2014 15:11:16 for ESAT by  

 1.8.7
